# Revealing hazard-exposure heterophily as a latent characteristic of community resilience in social-spatial networks

**DOI:** 10.1038/s41598-023-31702-9

**Published:** 2023-03-24

**Authors:** Chia-Fu Liu, Ali Mostafavi

**Affiliations:** Zachry Department of Civil and Environmental Engineering, Texas A&M University, 199 Spence St., College Station, TX 77843-3136 USA

**Keywords:** Natural hazards, Environmental social sciences

## Abstract

We present a latent characteristic in socio-spatial networks, hazard-exposure heterophily, to capture the extent to which populations with dissimilar hazard exposure could assist each other through social ties. Heterophily is the tendency of unlike individuals to form social ties. Conversely, populations in hazard-prone spatial areas with significant hazard-exposure similarity, homophily, would lack sufficient resourcefulness to aid each other to lessen the impact of hazards. In the context of the Houston metropolitan area, we use Meta’s Social Connectedness data to construct a socio-spatial network in juxtaposition with flood exposure data from National Flood Hazard Layer to analyze flood hazard exposure of spatial areas. The results reveal the extent and spatial variation of hazard-exposure heterophily in the study area. Notably, the results show that lower-income areas have lower hazard-exposure heterophily possibly caused by income segregation and the tendency of affordable housing development to be located in flood zones. Less resourceful social ties in hazard-prone areas due to their high-hazard-exposure homophily may inhibit low-income areas from better coping with hazard impacts and could contribute to their slower recovery. Overall, the results underscore the significance of characterizing hazard-exposure heterophily in socio-spatial networks to reveal community vulnerability and resilience to hazards.

## Introduction

Examining of interactions within socio-spatial networks embedded in communities and investigating social connections among disparate populations can illuminate subtle characteristics in community resilience assessments^1–5^. Networks are formed in social and spatial contexts and therefore cannot be explained solely by social or geographic characteristics^6^. Analyzing social structure with geographic information can extend our understanding of complex networks embedded in communities. In the field of community resilience, numerous vulnerability assessment techniques have been proposed to identify geographically hazard zones^7–9^. For instance, the spatial extent of hazard exposure can be computed by considering both the yearly average frequency of hazard (e.g. tropical cyclones) and exposed populations^10^. The intersection of socio-spatial network characteristics and spatial hazard exposure could reveal insights beyond the standard index-based approaches (such as social vulnerability index). This study reveals hazard-exposure heterophily as a latent characteristic in the socio-spatial networks of a community that could improve resilience and recovery. Heterophily refers to the tendency of groups of people to maintain a higher proportion of relations with members of groups other than their own^11^. In this study, we will characterize the heterophily in the social networks of a community based on the spatial extent of hazard exposure.

Standard index-based approaches for spatial characterization of community vulnerability and hazard exposure do not capture the dynamics of populations and places in socio-spatial networks, and hence provide a limited view of the extent of a community’s vulnerability and resilience. The identified hazard-exposure heterophily characteristic captures the extent to which residents in spatial areas exposed to natural hazards have social connections with residents outside hazard zones. The rationale is that social connections with non-hazard-prone areas are more resourceful during flood hazards. In the context of this study, resourcefulness refers to the ability to provide support and aid to impacted areas and is not limited to economic resources. For instance, people in non-hazard-prone areas have a better chance to provide emergency relief or temporary housing to their hazard-prone neighborhoods when experiencing a hazardous event. On the contrary, if residents of two spatial areas have strong social connectedness, but both experience flood impacts, they would probably not be resourceful to each other. In the context of this study, resourceful social ties are defined as social connections that can aid response and recovery from natural hazard impacts.

The existing literature has emphasized the importance of social cohesion and social capital in community resilience^12–16^. Researchers have proposed indices (such as social vulnerability index^17–20^ and social capital index^21,22^) to specify the spatial patterns of social capital^23,24^ and cohesion^15,16^ across communities. However, the existing approaches suffer from two limitations: first, they determine the extent of social cohesion/capital based on attributes (e.g., socio-economic characteristics) of a spatial area (e.g., census tract or ZIP code tabulation area (ZCTA)) rather than on measured social connections^17,25–27^. This limitation is due mainly to challenges in measuring social connections among populations of different spatial areas. This limitation can be overcome using emerging data sources such as social media platforms. Second, existing approaches do not account for the extent of resourcefulness of social links during hazards by considering all social ties as homogenous. Residents of two spatial areas which are both flooded probably would not have resourcefulness to help each other. These two limitations have hindered the ability to examine the intersection of hazard exposure and socio-spatial networks simultaneously in an integrated manner.

**Figure 1:**
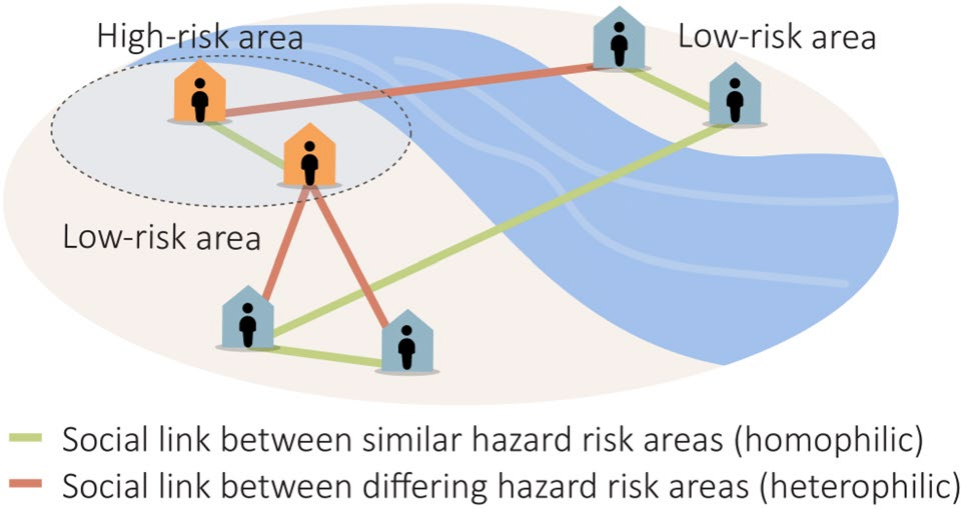
The illustration of *heterophily* and *homophily*.

In this study, we address this gap by examining hazard-exposure heterophily among spatial areas based on resourceful social ties (Fig. 1). In communities, social connections are created based on homophily (the opposite of the characteristic of heterophily), similarity of socio-demographic attributes, such as income and race^28,29^. When natural hazards occur, these social connections are leveraged for responding and recovering from impacts of the hazard^30^. The rationale here is that the social connections with residents outside of a hazard zone are more resourceful during hazard events^14^. On the other hand, if residents of two spatial areas have strong social connection, but with both experiencing similar hazard impacts, the extent to which these social connections could be leveraged diminishes. This characteristic in socio-spatial networks can be explained based on hazard-exposure heterophily. In network science, heterophily in a network is defined as the extent of dissimilarity among the attributes of nodes that have links^11,31^. In socio-spatial networks, a greater hazard-exposure heterophily indicates a greater extent of resourceful social connections available to the population of a spatial area to aid them during their response and recovery from the impacts of a natural hazard. In the following sections, the datasets and methodology for assessing hazard-exposure heterophily are presented.

## Data and methodology

Harris County, which encompasses the Houston, Texas, metropolitan area was selected as the study area to demonstrate the approach for examining hazard-exposure heterophily. Harris County is one of the largest metropolitan areas in the United States and also one of the most flood-prone regions in the world. Hence, we selected this study area to examine hazard-exposure heterophily in the context of flood hazard exposure. We constructed the socio-spatial network model of community by using ZCTAs to represent spatial area nodes *i* = 1,2,…, n. The ZCTA geodatabase was extracted from the selected state-based geographic and cartographic information from the U.S. Census Bureau’s Topologically Integrated Geographic Encoding and Referencing (TIGER) database. The geodatabases include feature class layers of information for the entire U.S.

To specify the level of hazard exposure of each spatial node (ZCTA), we focused on flooding as the primary hazard event in the study area. We collected 100-year and 500-year flood-hazard layer data from the Federal Emergency Management Agency (FEMA). FEMA provides the most recent nationwide extract of the National Flood Hazard Layer (NFHL) geospatial database through web-mapping services. This dataset includes flood map panel boundaries, flood hazard zone boundaries, and other information related to flood control zones and areas. The geospatial data was useful for calculating the floodplain area percentage for each ZCTA and thus determine the level of flood hazard exposure. In this study, we included both 100-year and 500-year layers as the floodplain layer. We classified ZCTAs into two groups according to the median of floodplain area percentage. ZCTAs with the highest 50% floodplain area percentage are labeled as high-flood-exposure ZCTAs (H), while ZCTAs listed on lowest 50% floodplain area percentage would be regarded as low-flood-exposure ZCTAs (L). Accordingly, links between ZCTAs with dissimilar hazard exposure attribute are designated as resourceful links and links between high-flood-prone areas are designated as non-resourceful links.

To specify the *links* or *ties* that connect each spatial area node in socio-spatial network, we used the Social Connectedness Index (SCI)^32^, which is based on friendship links on Meta, the company formerly known as Facebook, Inc. The SCI provided comprehensive measures of friendship networks at a national level. This dataset includes an aggregated and anonymized snapshot of all active Meta users; the relative frequency of Meta friendship links measures the intensity of connectedness between locations. Locations are assigned to users based on their information and activity on Meta, including the city of residence stated on their Meta profile, and device and connection information. In this study, SCI is presented at the ZCTA-level, which are pairs of five-digit ZCTA connections. Only ZCTAs with more than 500 residents and enough Meta users to produce meaningful estimates were included, leaving 26,271 of 33,139 ZCTAs covering 99.4% of the United States population. The measure of Social Connectedness ${e}_{i,j}$ between two ZCTAs *i* and *j* is

1 $${e}_{i,j}=\frac{{\nu }_{i,j}}{User(i)\times User(j)}$$

where $User(i)$ and $User(j)$ are the number of Meta users in locations *i* and location *j*, and ${\nu }_{i,j}$ is the number of Meta friendship connections between locations *i* and location *j*. For each value in the dataset, the measure was scaled to have a fixed maximum value by dividing the original measure by the maximum and multiplying by 10^9^ and the lowest possible value of 1. Each measure was rounded to the nearest integer.

### Methodology

To capture hazard-exposure heterophily, we defined the resourceful-tie rate ${\rho }_{i}$ (*i* = 1, 2,…, *n*) as a node attribute for each of ZCTA

2 $${\rho }_{i}=\frac{{\sum }_{j\in L}{e}_{i,j}}{{\sum }_{j=1}^{n}{e}_{i,j}}\times 100\%$$

The numerator is the summation of Social Connectedness that ZCTA *i* received from low-flood-exposure ZCTAs (*L*); meanwhile, the denominator is the total Social Connectedness that ZCTA *i* could receive from all its friendship ZCTAs (both *L* and *H*). The social tie a spatial area received from non-hazard-prone areas was regarded as more resourceful during hazard events. Therefore, the resourceful-tie rate ${\rho }_{i}$ was defined in a way to measure the percentage of resourceful social ties each ZCTA has. Notably, the resourceful-tie ${\rho }_{i}$ aims to capture the degree of hazard-exposure heterophily of hazard-prone areas but will also at the same time capture the hazard-exposure homophily of non-hazard-prone areas. Hazard-exposure heterophily exists in the friendship link which connects low-flood-exposure ZCTAs (*L*) with high-flood-exposure ZCTAs (*H*). On the other hand, a friendship link connecting two ZCTAs in the same group, which could be an *L*–*L* pair or an *H*–*H* pair, is said to display hazard-exposure homophily. This study will focus on the analysis of hazard-exposure heterophily in hazard-prone areas. By calculating the resourceful-tie rate for each node, we are able to quantify the degree of hazard-exposure heterophily shown in the social-spatial network. Table 1 shows the summarized variable description in the network model.

**Table 1:** Variable description of the network model.

Definition	Notation
Node of ZCTA	*i* = 1, …, *n*
Social connectedness	*e*_*i*, *j*_, *i*, *j* = 1, …, *n*
Number of Meta friendship connections	${\nu }_{i,j}$, *i*, *j* $\in$ *N*
Number of Meta users	*User*(*i*), *i* = 1, …, *n*
Low-flood-exposure ZCTAs	*L*
High-flood-exposure ZCTAs	*H*
Resourceful-tie rate	${\rho }_{i}$, *i* = 1, …, *n*

## Results

**Figure 2:**
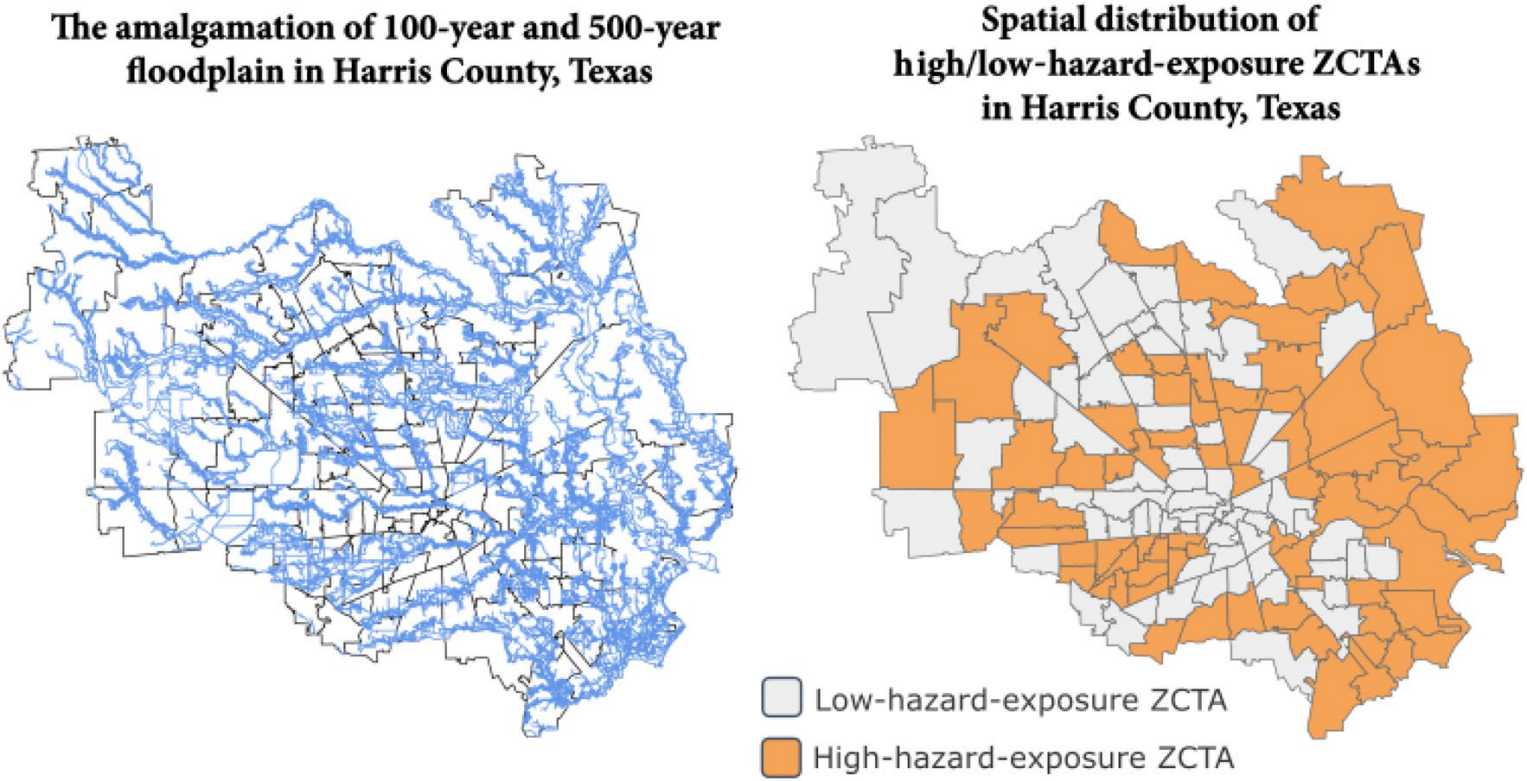
Left: The amalgamation of 100-year and 500-year floodplain in Harris County, Texas. Right: The classification of high-hazard-exposure ZCTAs (*H*) and low-hazard-exposure ZCTAs (*L*) in Harris County, Texas.

In this section, we discuss the results of hazard-exposure heterophily in the context of Harris County. First, we extracted the geographic data of 140 ZCTAs in Harris County from the TIGER geodatabase and created nodes representing the spatial network. Second, to measure the level of hazard exposure for the 140 ZCTAs, we combined both 100-year and 500-year flood hazard layers in Harris County and calculated the floodplain area percentage for each ZCTA (Fig. 2). Then, we divided the 140 ZCTAs based on the median of floodplain area percentage (28.77%) into a high-flood-exposure group (*H*) and a low-flood-exposure group (*L*). Third, we constructed the socio-spatial network by adding the Social Connectedness links and weights to the network. In total, Harris County contains 223 links among ZCTAs based on the Meta’s Social Connectedness Index.

**Figure 3:**
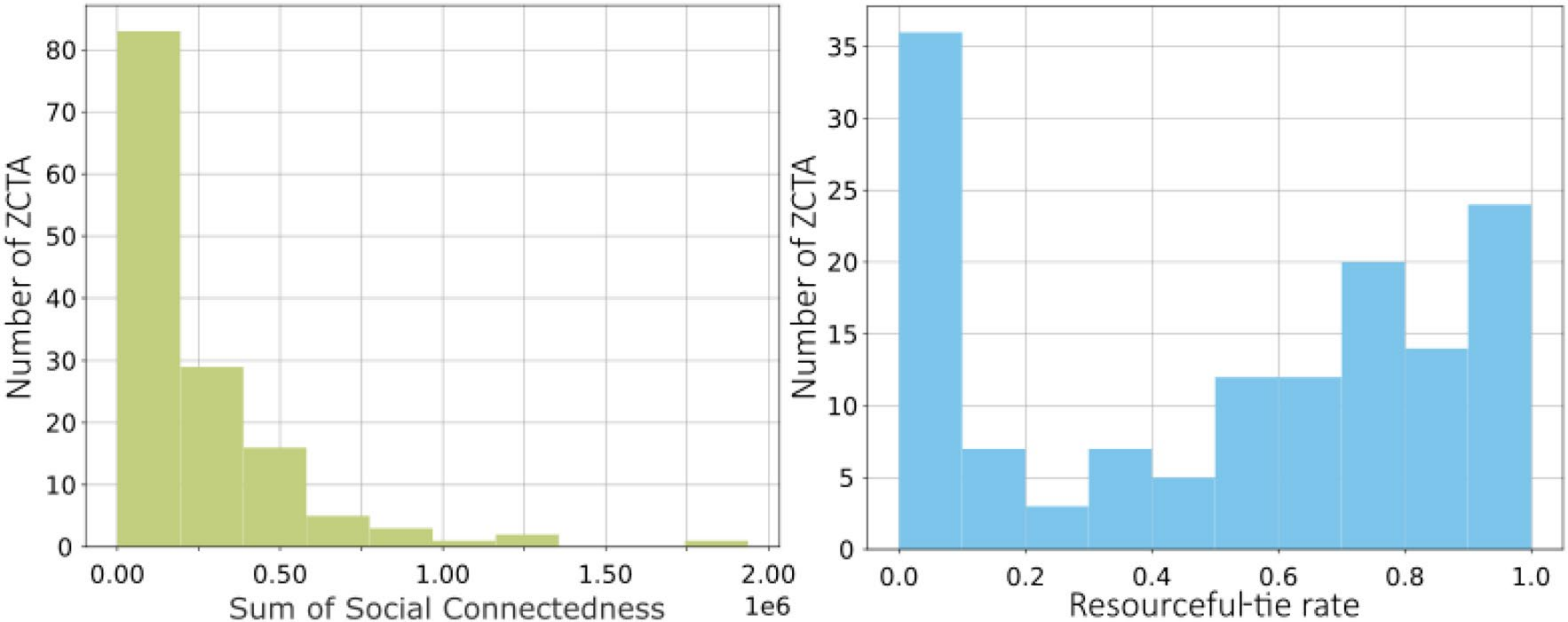
Left: The distribution of the Sum of Social Connectedness with skewness = 2.82. Right: The distribution of Resourceful-tie rate with skewness = − 0.26.

In the next step, we calculated the resourceful-tie rate ${\rho }_{i}$ for 140 ZCTAs and compared it with the summation of Social Connectedness ${\sum }_{j=1}^{n}{e}_{i,j}$, which measures the total friendship link of ZCTA *i*, to calculate the extent of the resourcefulness of each link among node pairs. In Fig. 3, the sum of Social Connectedness shows a right-skewed unimodality, while the resourceful-tie rate appears in a more bimodal shape. The bimodal distribution shown in the resourceful-tie rate indicates the disparity of resourceful connections among 140 ZCTAs.

**Figure 4:**
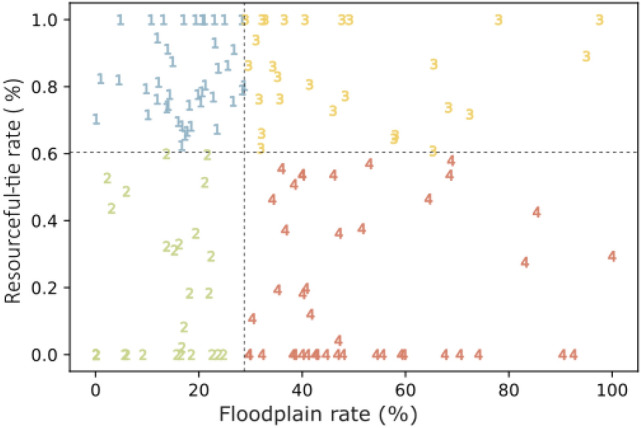
The classification of 140 ZCTAs in Harris County, Texas, according to Floodplain rate and Resourceful-tie rate.

**Figure 5:**
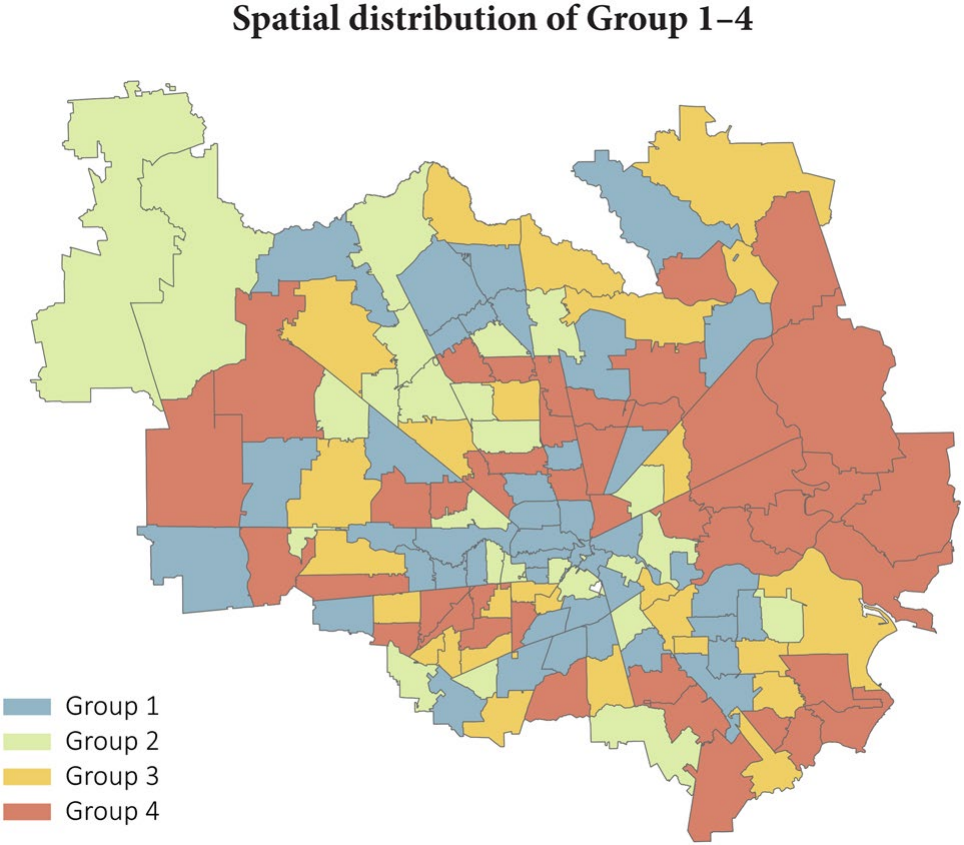
The spatial distribution of Group 1–Group 4.

In the second step, 140 ZCTAs were classified into four groups based on two attributes: floodplain rate and resourceful-tie rate (as shown in Figs. 4 and 5). We selected the median floodplain rate (28.77%) and the median resourceful-tie rate (60.37%) to be the cutoff points for both classifications. ZCTAs with floodplain rates less than 28.77%, fall into Group 1 (blue) and Group 2 (green), including the low-flood-exposure ZCTAs (*L*). Group 3 (yellow) and Group 4 (red) include high-flood-exposure ZCTAs (*H*) (those with a floodplain rate $\ge$ 28.77%). Among the high-flood-exposure ZCTAs, Group 3 has greater hazard-exposure heterophily as indicated by the higher resourceful-tie rate. Hence, ZCTAs in Group 3 would have more resourceful links that could aid them during flood events, ostensibly enabling them to cope with the impacts and for a faster recovery. On the other hand, the lower resourceful-tie rate in Group 4 indicates hazard-exposure homophily, which would reduce the ability of impacted residents in those areas to aid each other during flood events.

Third, we evaluated the disparity in resourceful links in Group 3 and Group 4 with respect to their socio-economic information (shown in Table 2). We applied the statistical hypothesis test to the difference of median household income between Group 3 and Group 4. The result of the t-test shows that the means of median household income between Group 3 ($90.01 K) and Group 4 ($67.61 K) are significantly different (*p*-value = 0.026) with $$\alpha =0.05$$ from each other. This implies that ZCTAs in Group 4 that show a lower degree of hazard-exposure heterophily, and thus a higher degree of homophily, are mainly from lower income groups. This result is significant in two aspects: first, the results reveal one mechanism causing lower-income areas recover more slowly after flood events. A number of studies^33,34^ have reported the slow recovery of low-income areas after flood events in Harris County. The result presents one mechanism that negatively affects such slow recovery due to hazard-exposure homophily. Second, this result highlights the effect of income segregation and community development patterns (locating affordable housing developments in flood zones^35,36^) on community resilience. The homophily among spatial areas with similar income levels when intersecting with the hazard-exposure landscape would create a hazard-exposure homophily among these low-income areas which negatively affects their ability to cope and recovery from flood events.

**Table 2:** The mean of household income median for Group 1–Group 4.

Group	Mean of household income median (in thousand)
1	$85.30
2	$90.01
3	$90.44
4	$67.61

## Discussion

In this study, hazard-exposure heterophily is introduced as a latent characteristic of socio-spatial networks that affects community resilience. Hazard-exposure heterophily captures the extent to which social connection among populations living in spatial areas with similar/dissimilar hazard exposure could be resourceful during hazard events, thus mitigating disaster impacts. The main idea in this study departs from the existing approaches in hazards/disaster research that consider the extent of social connections and hazard exposures in isolation and fail to capture the intersection of socio-spatial networks and hazard exposure in assessing community vulnerability and resilience. This study addresses this gap by revealing hazard-exposure heterophily, which is a latent characteristic determining the extent of resourcefulness of social connections among spatial areas based on their respective hazard exposure.

This study leverages a unique social connectedness dataset based on Meta’s Social Connectedness Index that provides a novel measure of social connections among residents of different spatial areas. This data is used in conjunction with the flood exposure data to construct a socio-spatial network model of the community from which can be determined the extent of resourceful links associated with residents of each spatial area. Accordingly, a hazard-prone spatial area with low resourceful links can be identified and characterized as having a low-hazard-exposure heterophily (or high-hazard-exposure homophily). The findings reveal the spatial variation of hazard-exposure heterophily. In addition, the findings reveal that low-income areas have lower hazard-exposure heterophily (or greater hazard-exposure homophily). The theoretical significance of this finding can be viewed from two aspects: first, this finding reveals one possible mechanism that hinders low-income areas from coping and recovering from flood impacts. The literature^33,34,37^ provides strong evidence about greater impacts and slower recovery of low-income areas during flood events and has attributed the greater impacts and slower recovery to socio-economic characteristics^38–40^. The finding in this study however, reveals that hazard-exposure homophily (or lack of heterophily) reduces the number of resourceful social links available to low-income areas, and thus could negatively affect their resilience and recovery. Second, these findings uncover that income segregation in communities (i.e., residents with similar income status have strong social ties), would lead to hazard-exposure homophily in low-income areas, and subsequently adversely affect access to resourceful social ties. This result is consistent with the reported income segregation in cities and suggests that income segregation reinforces hazard-exposure homophily and thus reduces the resourceful ties that low-income that can increase residents' need resilience and quicken recovery. Previous studies^35,36^ have reported the negative effects of income segregation on communities; however, little theoretical explanation exists regarding the mechanisms by which income segregation affects community resilience and recovery. The finding from this study in the context of Harris County suggests that income segregation creates hazard-exposure homophily, and thus, negatively affects access to resourceful social ties during hazard events. Furthermore, this study’s outcomes open up new lines of inquiry for future studies to adopt the hazard-exposure heterophily characteristic and measures to explore the relationship between hazard-exposure heterophily and various aspects of community recovery and resilience. Accordingly, this study and findings contribute to and inform future research for a better understanding of community resilience mechanisms at the intersection of socio-spatial networks and hazard exposure characteristics based on leveraging emerging datasets.

From a practical perspective, the specification of the spatial variation of hazard-exposure heterophily could complement the existing index-based approaches (such as the social vulnerability index and social capital index) to inform hazard mitigation, and response and recovery plans and actions. For example, the spatial areas with a lower number or degree of resourceful links can be prioritized by response and relief agencies and public officials for resource allocation and recovery assistance as they lack extensive resourceful social links.

Although this study leveraged social connectedness in conjunction with the flood exposure data to provide a novel measurement of community resilience, our choice of data also showed two shortcomings. First, the Social Connectedness Index (SCI) only provides aggregate and anonymized friendship data of Meta users. The SCI may not capture social ties that are not captured in social media. Second, this study selected National Flood Hazard Layer (NFHL) as an indicator for hazard-exposure assessment in a general way rather than based on a real-world event. Based on the initial implementation presented in this study, future research can build upon this flexible socio-spatial framework and examine hazard-exposure heterophily for different scenarios of hazards.

## Data Availability

The data that support the findings of this study are available from Meta Social Connectedness Data, but restrictions apply to the availability of these data. The data can be accessed upon request submitted on Meta Data for Good Program. Other data we use in this study are all publicly available.

## Code availability

The code that supports the findings of this study is available from the corresponding author upon request.
